# A Rare Case of Bare Lymphocyte Syndrome Presenting As Chronic Type 2 Respiratory Failure

**DOI:** 10.7759/cureus.64951

**Published:** 2024-07-19

**Authors:** Aayushi Bhatnagar, Vivek R Velagala, Jayant D Vagha, Sham Lohiya, Ajinkya Wazurkar, Shailesh Wandile, Chaitanya Kumar Javvaji

**Affiliations:** 1 Medicine, Jawaharlal Nehru Medical College, Datta Meghe Institute of Higher Education and Research, Wardha, IND; 2 Pediatrics, Jawaharlal Nehru Medical College, Datta Meghe Institute of Higher Education and Research, Wardha, IND

**Keywords:** hypercapnic respiratory failure, malnutrition, collapse of one lung, pulmonary consolidation, whole-genome sequencing, severe hypercapnia, airway intubation, atopy, central hypoventilation syndrome, bare lymphocyte syndrome

## Abstract

Type 2 respiratory failure, or hypercapnic respiratory failure, is brought on by low oxygenation (hypoxemia) and inadequate breathing (hypercapnia). It is produced by factors that can create an imbalance between the requirement and capacity of the respiratory system. The factors can include an increased requirement for muscles of respiration, reduction in their strength or effectiveness, or impediment of the ventilatory drive. Rarely, it can be caused by the bare lymphocyte syndrome (BLS), which usually affects young children and has a poor prognosis with accompanying debilitating disabilities. This is a case report that shares the unique findings of a 13-year-old patient with type 1 BLS and atopy, who is suffering from type 2 respiratory failure. She is susceptible to respiratory tract infections and has been treated for bronchopneumonia and tuberculosis in the past. She has been on assisted ventilation for the past 3.5 months, along with supplementary nutrition. She has been evaluated meticulously and methodically, ruling out other causes of her respiratory failure. Recognizing the root cause aided in her therapy and preventing mortality. This has been determined using clinical findings, lab results, and radiological reports. The diagnosis of hypercapnic respiratory failure was confirmed via an arterial blood gas analysis, whereas that of BLS was confirmed through a whole genome sequence test. Management entailed addressing the underlying cause, optimizing ventilation, and using mechanical ventilation to maintain respiratory function. Early detection and timely intervention were critical in enhancing the outcome for the patient.

## Introduction

Respiratory failure occurs when the respiratory process fails to perform either or both of its gaseous exchange operations. It is an important contributor to morbidity and mortality in intensive care units. It can be due to lung failure, which leads to hypoxemia, or it can be due to failure of the cardiac pump, resulting in hypercapnia and alveolar hypoventilation [1]. The ventilation/perfusion (V/Q) ratio determines the partial pressures of oxygen (O2) and carbon dioxide (CO2) within each alveolus, as well as the flow of blood through the capillaries. As the V/Q ratio declines, the partial pressure of oxygen lowers, and carbon dioxide increases in the circulation, leaving the alveolus. The opposite phenomenon occurs as the V/Q ratio rises. Pathologic processes affecting the lungs' airways, parenchyma, and vasculature can generate an imbalance in ventilation and perfusion, resulting in abnormal V/Q [2]. Ventilatory failure, also known as type 2 respiratory failure, is characterized by an increase in arterial partial pressure of carbon dioxide (PaCO2) > 45 mmHg, a reduction in the arterial partial pressure of oxygen (PaO2) < 60 mmHg, and a decreased pH < 7.35. These changes are brought about by lung failure and/or raised carbon dioxide generation [3]. Its onset may be acute, insidious, or acute superimposed on chronic hypercarbia. The constant factor in all these situations is decreased alveolar ventilation for the amount of CO2 generated. This is usually seen in patients experiencing labored breathing due to airflow obstruction, poor pulmonary compliance, neuromuscular illness, or central respiratory failure, resulting in a reduced respiratory drive. It may also be caused by raised carbon dioxide generation from increased metabolism in cases of sepsis, fever, burn, or overfeeding [4,5]. Bare lymphocyte syndrome (BLS) is a condition that can sometimes present with type 2 respiratory failure. BLS is a rare genetic disorder of the immune system marked by a lack of specialized immune proteins in cells, particularly in lymphocytes. This results in immunodeficiency in the affected individuals, making them vulnerable to pathogenic invasion by microbes. BLS is a very rare disorder with scanty prevailing literature on the same. Only about 30 people have been reported with the syndrome so far. Most BLS patients experience recurring bacterial infections in their lungs and airways since early childhood. These recurring infections can produce bronchiectasis, a disease that destroys the bronchi and causes difficulty breathing [6].

## Case presentation

A 13-year-old female presented with chief complaints of generalized weakness, fever, and decreased appetite for three days. The child was a result of a non-consanguineous marriage and was born to a second gravida mother. As narrated by the mother, the child was asymptomatic three days ago when she started experiencing a decrease in appetite that was accompanied by weakness. The patient also developed a fever that was insidious in onset, mild grade, and was relieved with medication. There was no history of weight loss or evening rise in temperature. A month prior to admission, the child reportedly developed a cough for which she was taken to a local practitioner and was diagnosed with pulmonary tuberculosis (TB). After that, she was started on anti-tubercular therapy (ATT) with isoniazid (50 mg), rifampicin (75 mg), pyrazinamide (150 mg), and ethambutol (100 mg). On following up with a cartridge-based nucleic acid amplification test (CBNAAT) during her current hospital stay, she tested negative for *Mycobacterium tuberculosis* (MTB). The patient also had a history of having bronchopneumonia four years back, for which she was admitted for a month and subsequently treated. The child was a full-term normal delivery (FTND) with a birth weight of 3.5 kg. There were no noteworthy prenatal or perinatal incidents, and no history of neonatal intensive care unit (NICU) stay. She achieved all developmental milestones in due time and was vaccinated as per the national immunization schedule (NIS).

On examination after admission, the child had tachycardia with a heart rate (HR) of 142/min, respiratory rate of 20/min, saturation of peripheral oxygen (SpO2) of 82%, and was found gasping during sleep. According to the Indian Academy of Pediatrics (IAP), she was graded as having grade 3 malnutrition. Weight for age is 55% (less than the third percentile) and height for age is 93% (at the 10th percentile). The expected weight is 43 kg, and the expected height is 157 cm. There was decreased left-sided air entry with a progressive reduction of activity and the development of a drowsy sensorium. Normal heart sounds were auscultated along with a hyperdynamic apical impulse seen in the fifth intercostal space (ICS) lateral to the mid-clavicular line (MCL). Parasternal heave and gallop rhythm were present. The abdomen was soft and non-tender, with no organomegaly. On admission, the child was started on nasopharyngeal oxygen (NPO) therapy in view of shallow respiratory efforts and decreased SpO2. She was given intravenous (IV) fluids, and IV ceftriaxone (150 mg/kg/day), pantoprazole (1 mg/kg/day), and ondansetron (15 mg/kg/day) were administered. Tablet valproate (20 mg/kg/day) was given, and IV vancomycin (20 mg/kg) and IV azithromycin (10 mg/kg) were added. Mechanical ventilation was required on the sixth day due to frequent apneic episodes and respiratory acidosis (pH = 7.19, pCO2 = 90.6, pO2 = 43.8), for which the patient was intubated with an endotracheal tube (ET). Two days later, the patient was found gasping with an end-tidal volume (VTE) mismatch, and viscous secretions were suctioned out. The patient was re-intubated, and oxygen saturation was maintained in synchronized intermittent mandatory ventilation (SIMV) mode. Chest physiotherapy was started, and the sepsis screen and blood culture were negative, ruling out secondary causes of apnea. A normal magnetic resonance imaging (MRI) excluded any spinal abnormality, a normal computed tomography (CT) brain contrast ruled out any obvious brain parenchyma pathology, a sniff test ruled out diaphragmatic palsy, and a normal muscle biopsy and nerve conduction velocity report ruled out neuromuscular diseases like congenital myopathy or myasthenia gravis. Her laboratory investigation findings are illustrated in Table 1. 

**Table 1:** Laboratory investigations RBC: Red blood cell; WBC: White blood cell; ALP: Alkaline phosphatase; ALT: Alanine aminotransferase; AST: Aspartate transaminase; ELISA: Enzyme-linked immunosorbent assay

Serial number	Investigation	Value	Normal range	Interpretation
Complete blood count (CBC) investigations				
1	RBC count	5.34 million cells/cmm	4.0-5.4 million cells/cmm	Within normal limits
2	WBC count	7,600 cells/cmm	4,000-10,000 cells/cmm	Within normal limits
3	Platelet count	88,000 cells/cmm	150,000-400,000 cells/cmm	Decreased
Kidney function test (KFT)				
4	Urea	23 mg/dL	19-44.1 mg/dL	Within normal limits
5	Creatinine	1.2 mg/dL	0.72-1.25 mg/dL	
6	Sodium (Na^+^)	143 mmol/L	136-145 mmol/L	
7	Potassium (K^+^)	4.1 mmol/L	3.5-5.1 mmol/L	
Liver function test (LFT)				
8	ALP	89 U/L	24-147 U/L	Within normal limits
9	ALT	76 U/L	10-130 U/L	
10	AST	26 U/L	10-34 U/L	
11	Total protein	6.5 g/dL	5.2-8.2 g/dL	
12	Albumin	3.9 g/dL	2.4-4 g/dL	
13	Total bilirubin	0.3 mg/dL	0-0.8 mg/dL	
C-reactive protein (CRP)				
14	CRP	0.524 mg/dL	0.3-1.0 mg/dL	Within normal limits
Erythrocyte sedimentation rate (ESR)				
15	ESR	10 mm/hour	< 20 mm/hour	Within normal limits
D-dimer protein				
16	D-dimer	186 mg/mL	< 243 mg/mL	Within normal limits
Anti-nuclear antibodies (ANA) by ELISA method				
17	ANA	0.630 U	= 1.0 U: negative; 1.0-2.9 U: weakly positive; 3.0-5.9 U: positive; >/= 6.0 U: strongly positive	Negative
Adenosine deaminase (ADA)				
18	ADA	3.077	0-40 U/L	Within normal limits

**Figure 1:**
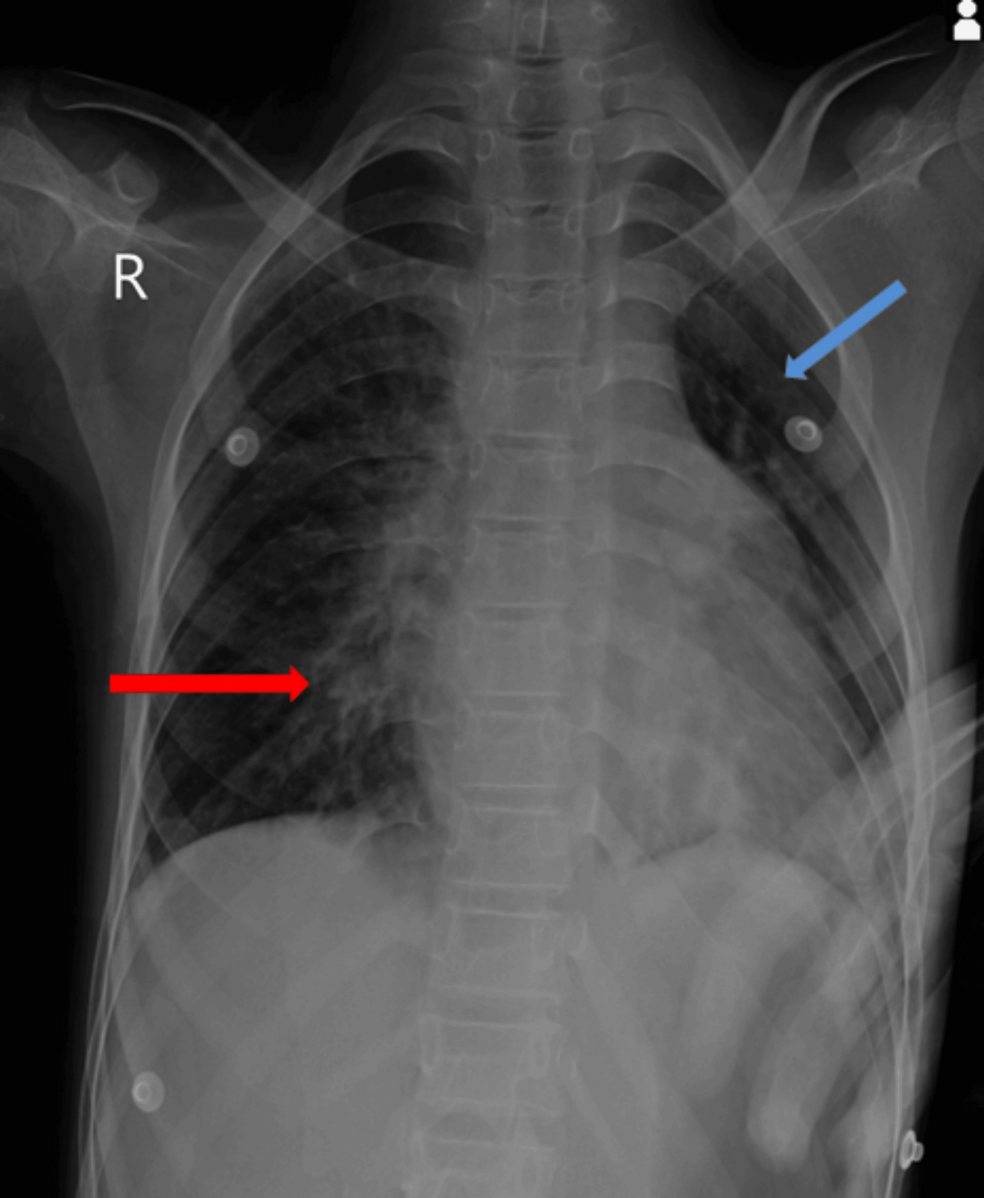
Chest X-ray showing right lung consolidation (indicated by red arrow) and left lung collapse (indicated by blue arrow)

A chest X-ray revealed left lung collapse and right lung consolidation as is shown in Figure 1.

**Figure 2:**
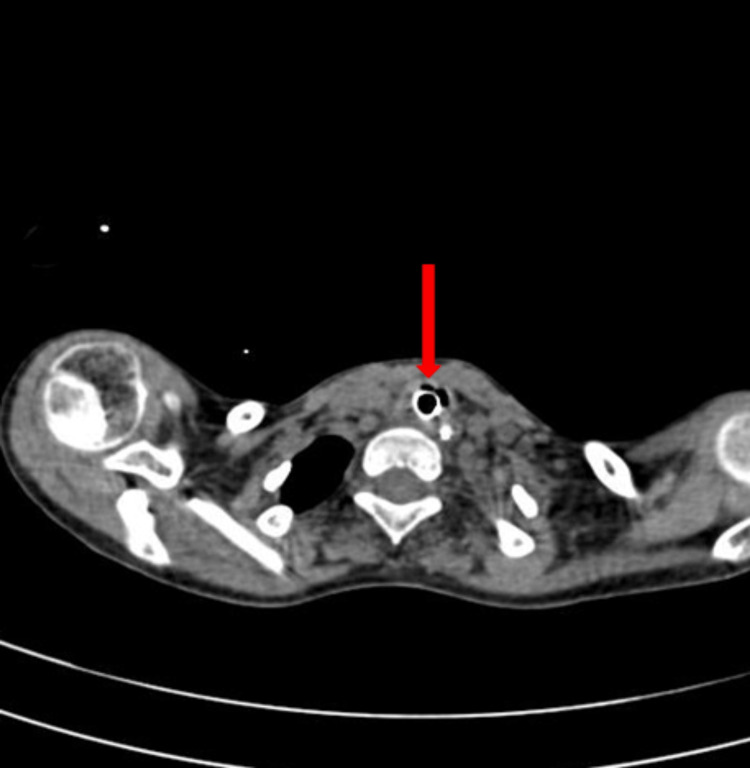
HRCT thorax showing tracheal deviation from the midline to the left side (indicated by the red arrow) HRCT: High-resolution computed tomography

**Figure 3:**
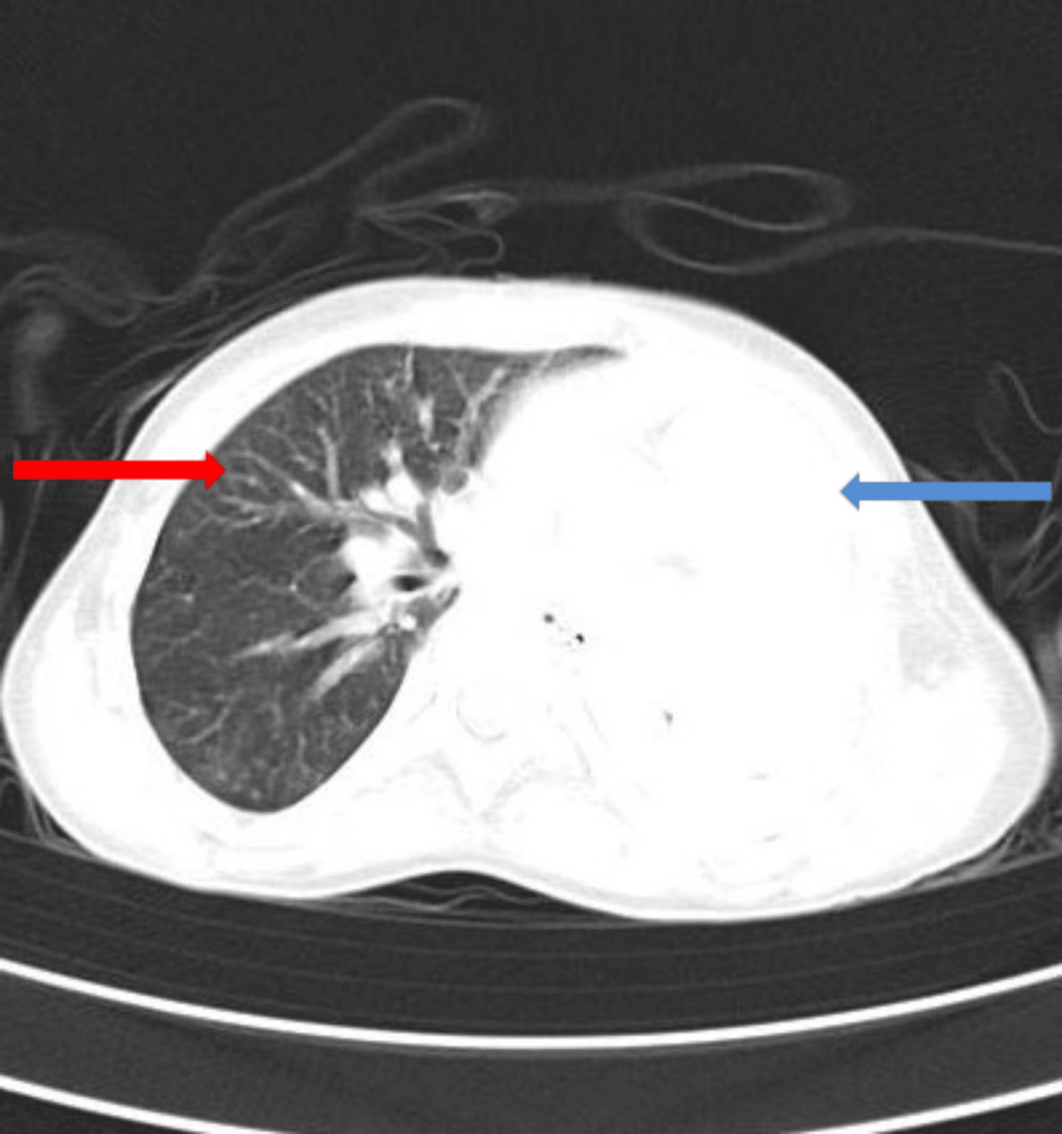
HRCT thorax showing left collapse (indicated by the blue arrow) and right lung consolidation with tree-in-bud appearance (indicated by the red arrow) HRCT: High-resolution computed tomography

A high-resolution computed tomography (HRCT) thorax showed a mediastinal shift towards the left side, as shown in Figure 2, left lung collapse, and consolidation in the right hemithorax with ground glass opacity and centrilobular nodules giving a tree bud appearance, as shown in Figure 3.

**Figure 4:**
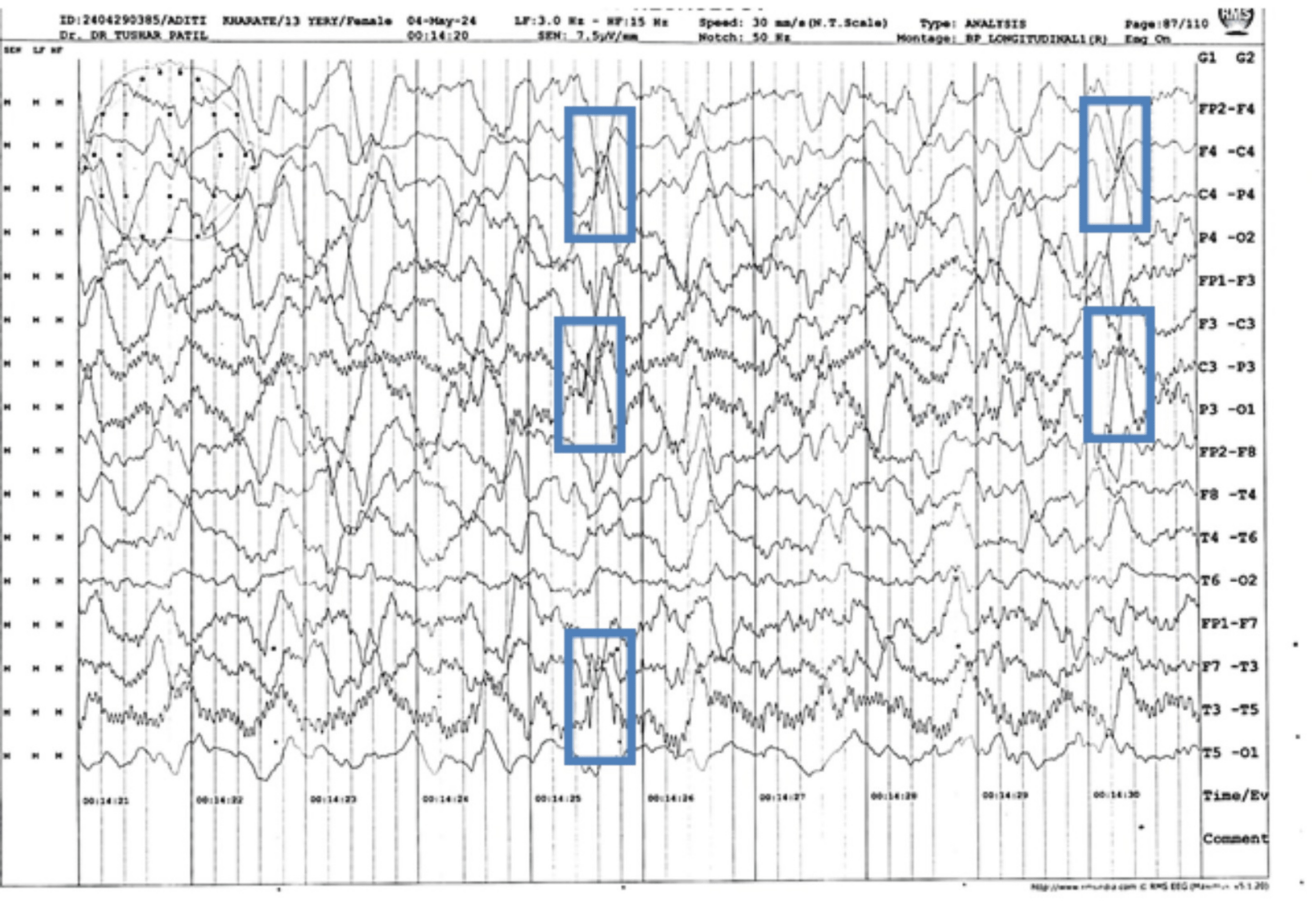
EEG reading showing sharp, high-amplitude waves as indicated by the blue frames EEG: Electroencephalogram

An electroencephalogram (EEG) was done, which showed abnormal intermittent sharp and high amplitude waves suggestive of generalized epileptiform activity, as shown in Figure 4.

The whole genome sequence test report ruled out central hypoventilation syndrome (PHOX2B gene mutation negative) and revealed atopy and BLS, type 1.

## Discussion

A disparity between the stress on respiratory muscles and their ability to cope, or the existence of insufficient ventilatory drive, results in hypercapnia. Alveolar hypoventilation can be caused by decreased minute ventilation or a poor response to a low V/Q ratio. While a sudden increase in PaCO2 is linked to respiratory acidosis, chronic persistence of this condition may lead to metabolic compensatory mechanisms coming into play and rectifying the respiratory acidosis [6]. In this case, respiratory acidosis persists along with raised PaCO2 even three months into the treatment. This case is unique because of its rare presentation and evasive etiology. The existing literature on type 2 respiratory failure tells us about the various diseases that can present with hypercapnia, such as abnormalities with central respiratory drive, spinal cord abnormalities including kyphoscoliosis, motor nerve abnormalities, obesity, neuromuscular illnesses like cervical cord lesions, Guillain-Barre syndrome, myasthenia gravis, and muscular dystrophy, airway and lung abnormalities including COPD, blocked airways, and bronchiectasis, and increased CO2 generation [7-9]. A wide array of clinical, biochemical, and radiological investigations ruled out all of these conditions. Our patient complained of disturbed sleep due to sleep apnea, which is a common manifestation of type 2 respiratory failure. Hypoventilation during sleep is common in slowly progressive illnesses associated with increasing ventilatory dysfunction, and it can occur months or years before wakeful respiratory failure, subject to the rate of evolution of the underlying ailment. The primary explanation for the increased susceptibility to hypoventilation during sleep is the decreased ventilatory drive, particularly during rapid eye movement (REM) sleep. Indeed, the consequence of a mismatch between demand and capacity may first be noticeable only during REM sleep. As the condition progresses, hypoventilation becomes more noticeable in other sleep stages [10].

The case was initially managed with the differential diagnosis of central hypoventilation syndrome in mind, which was later ruled out by genetic testing. The test revealed atopy and BLS, type 1. Atopy is a disorder characterized by immunological susceptibility to a variety of antigens or allergens, resulting in immunoglobulin E (IgE) overproduction. As a result, hypersensitive reactions to allergens, such as environmental proteins, which are ordinarily harmless and are primarily found in plant pollen and home dust, occur. The most prevalent symptoms of atopy include bronchial asthma, rhinitis, atopic dermatitis, eczema, and food allergies [11]. BLS can be caused by a deficiency in any of the four genes that code for the regulatory factors required for major histocompatibility complex class II (MHC-II) gene function. A mutation in any of the four genes causes a comparable clinical condition. Frequent digestive system infections, pneumonia, and bronchitis, as well as acute septicemia, are common manifestations. Infections typically begin in the first year of life and develop rapidly, often resulting in mortality before the age of 10 [12]. There is no known existing literature on the prevalence of BLS in the adolescent age group, which makes this case unique. This case also sheds light on the poor quality of life that a patient with BLS has due to its various clinical manifestations, making it essential to diagnose and promptly treat the condition at the earliest. The child continues to be managed by a spontaneous breathing trial (SBT) using a t-piece during the day and continuous positive airway pressure (CPAP) during the night. There is no gasping, distress, or altered sensorium. Multivitamins, including zinc, calcium, and vitamin D, are administered. The patient is given nebulization with mucomix at 12-hour intervals, and suctioning is done periodically [13].

## Conclusions

Diagnosing the underlying cause of type 2 respiratory failure in this patient was based on exclusion by clinical evaluation, biochemical investigations, and radiological reports, and confirmed by studying the genomic sequence. BLS can present a variety of ailments, which can be a challenge for the diagnostician. This case illuminates the importance of following a protocol and investigating thoroughly to eliminate the differentials and approach a diagnosis. The patient was managed by maintaining her airway through intubation initially, followed by CPAP. Suctioning was done, and antibiotics were administered to prevent infection. She was given fluids, fed through the Ryles tube, and multivitamins to maintain her electrolyte balance and improve her nutritional status. A combination of prompt response to respiratory distress and careful monitoring helped alleviate her symptoms and restore function.
